# Unraveling the genetic origin of ‘Glera’, ‘Ribolla Gialla’ and other autochthonous grapevine varieties from Friuli Venezia Giulia (northeastern Italy)

**DOI:** 10.1038/s41598-020-64061-w

**Published:** 2020-04-29

**Authors:** Manna Crespan, Daniele Migliaro, Simone Larger, Massimo Pindo, Carlo Petrussi, Marco Stocco, Denis Rusjan, Paolo Sivilotti, Riccardo Velasco, Erika Maul

**Affiliations:** 1 CREA Research Centre of Viticulture and Enology, Conegliano, Treviso Italy; 2 Unit of Computational Biology, Research and Innovation Centre, Fondazione Edmund Mach, San Michele all’Adige, Trento, Italy; 3 Viticulturist, Cividale del Friuli, Udine, Italy; 4 ERSA, Agenzia Regionale per lo Sviluppo Rurale, Pozzuolo del Friuli, Udine, Italy; 5 Department of Agronomy, Biotechnical Faculty, University of Ljubljana, Ljubljana, Slovenia; 6 Department of Agricultural, Food, Environmental and Animal Sciences, University of Udine, Udine, Italy; 7 JKI - Institute for Grapevine Breeding Geilweilerhof, Siebeldingen, Germany

**Keywords:** Genotype, Plant genetics

## Abstract

‘Glera’ and ‘Ribolla Gialla’ are the most economically relevant local grapevine cultivars of Friuli Venezia Giulia region (north-eastern Italy). ‘Glera’ is used to produce the world-renowned Prosecco wine. ‘Ribolla Gialla’ cultivation is constantly increasing due to the strong demand for sparkling wine and is the most important variety in Brda (Slovenia). Knowledge of local varieties history in terms of migration and pedigree relationships has scientific and marketing appeal. Following prospections, genotyping and ampelographic characterization of minor germplasm in Friuli Venezia Giulia, a further research was developed to understand the parentage relationships among the grapevine varieties grown in this region. An integrated strategy was followed combining the analysis of nuclear and chloroplast microsatellites with the *Vitis* 18k SNP chip. Two main recurrent parents were found, which can be regarded as “founders”: ‘Vulpea’, an Austrian variety parent-offspring related with at least ten Friuli Venezia Giulia cultivars, among them ‘Glera’, and ‘Refosco Nostrano’, first degree related with other six Friuli Venezia Giulia varieties. ‘Ribolla Gialla’ was shown to be another member of the impressively long list of offspring derived from the prolific ‘Heunisch Weiss’. Combining molecular markers and historical references was a high-performance strategy for retracing and adjusting the history of cultivars.

## Introduction

Dealing with local grapevine varieties history is easier nowadays than in the past, due to the availability of molecular tools. Genotyping performed with SSR (Simple Sequence Repeats) markers supports this kind of study, increasing knowledge on grapevine biodiversity, varieties migration and pedigree relationships. In this respect, many parents of traditional cultivars are still missing and difficult to find, because some ancestors may be lost due to genetic erosion. The agreement on a standard set of nine SSR-markers and the growing availability of genetic fingerprints has documented germplasm exchange not only among neighboring regions, but also among very distant countries in previously unknown detail. Indeed, the molecular approach does not need comparisons with selected varieties and allows easy massive data exchange and the use of all available genotyping information^1^.

The main varieties grown in Friuli Venezia Giulia (FVG) region, North-East Italy, are ‘Pinot Gris’ (7,228 ha) and ‘Glera’ (5,861 ha), followed by ‘Merlot’ (2,058 ha), ‘Tocai Friulano’ (1,516 ha), ‘Chardonnay’ (1,355 ha), ‘Sauvignon Blanc’ (1,326 ha) (http://catalogoviti.politicheagricole.it) and ‘Ribolla Gialla’ (1,159 ha, unpublished; data extracted from Friuli Venezia Giulia regional vineyard database). Beyond minor and already known local varieties^2^, prospections of grapevine germplasm in FVG evidenced the presence of previously unknown varieties^3,4^.

The most economically relevant local cultivars in FVG are ‘Glera’, used to produce the world-renowned Prosecco wine, and the ancient, recently re-discovered, ‘Ribolla Gialla’. ‘Glera’ DOP (Protected Denomination of Origin) area expanded from Veneto to FVG, for a total of about 25,000 ha; nowadays ‘Glera’ covers 3.9% of the Italian wine-growing area (https://www.enolo.it/glera-veneto-ettari-proseccco-doc). The land cultivated with ‘Ribolla Gialla’ in Italy was really limited in 1982 (93 ha), but constantly increased reaching 284 ha in 2000, 435 ha in 2010 (http://catalogoviti.politicheagricole.it), and overcame 1,000 ha in the last years due to the strong demand for sparkling wine on the international market. ‘Ribolla Gialla’ sparkling wines are highly appreciated by consumers, forecasting a further increase of the cultivated area in the near future. ‘Rebula’ (‘Ribolla Gialla’) in Brda (Slovenian Collio) is the traditional and most important grapevine variety, nowadays covering 20% of the entire viticulture area. The first reliable mention of the variety by a priest Matija Vertovec dates to 1844 in a book Vinoreja^5^ and it has been the subject of several studies regarding genotyping and potential parentages and pedigree, without consistent reports^6–9^. Furthermore, the first mention of Ribolla of Rosazzo wine in Cividale del Friuli (northeastern Italy) dates to 1409^10^.

Although parentage analysis appeared to be suitably tackled using microsatellites, some problems persisted. Analyzing dozens of microsatellites required a great deal of effort in selecting the best markers, optimizing PCR conditions, scoring and calling alleles. More recently, with the availability of genomic resources and multiplexed methods to assay many single nucleotide polymorphisms (SNPs) simultaneously, researchers have turned to SNP approaches.

In this study, an integrated strategy was followed combining the analysis of 12 nuclear and 8 chloroplast microsatellites with the *Vitis* 18k chip for grapevine genotyping, comprising 18,071 SNPs selected in 47 *V. vinifera* genotypes and 18 non-*vinifera Vitis* species^11^. This tool is expected to outperform SSRs because it inspects thousands of points in one analysis and overcomes the slow informative power of single SNPs, which are bi-allelic.

## Materials and methods

### Plant material

Seventy-nine grapevine accessions from different repositories in Italy, Germany and Slovenia were analyzed (Table S1). The samples included varieties cultivated in FVG and in neighboring areas, and three pairs of already known trios, used as references to evaluate the threshold values obtained for data elaboration: ‘Raboso Veronese’ = ‘Raboso Piave’ × ‘Marzemina Bianca’^12^, ‘Vitouska’ = ‘Malvasia Bianca Lunga’ and ‘Glera’ (*alias* ‘Prosecco Tondo’)^13^, and ‘Manzoni Bianco’ = ‘Pinot’ × ‘Riesling’^14,15^. Six accessions belonging to ‘Heunisch Weiss’ variety were analyzed, some of them being somatic variants^16^: ‘Heunisch Weiss’, ‘Heunisch Weiss Seedless’, ‘Heunisch Dreifarbig’, ‘Heunisch Rotgestreift’, ‘Rebula Stara’ and ‘Liseiret’; they came from three countries; Germany, Italy (Piedmont, Northwest Italy) and Slovenia. Two ‘Ribolla Gialla’ accessions were genotyped, one from the Italian Collio and the other from the Slovenian Brda.

### Genomic DNA extraction

Genomic DNA was extracted from young freeze-dried leaves harvested from 79 accessions, using the QIAGEN DNeasy 96 Plant Kit (QIAGEN GmbH, Hilden, Germany) according to the manufacturer’s protocols with small modifications: 1.6% PVP40 (Sigma Aldrich) was added to the AP1 buffer and samples were incubated at 65 °C for 5 minutes; DNA was eluted in milliQ water at 65 °C. DNA quantification was performed using Quant-iT™ PicoGreen™ dsDNA Assay Kit (ThermoFisher Scientific) by Synergy2 Fluorometer (Biotek); DNA quality was checked both on an Agilent 2200 Tapestation (Agilent Technologies, CA), using the DNA genomic ScreenTape (Agilent Technologies) for DNA integrity detection, and NanoDrop 8000 Spectrophotometer (Thermo Scientific, MA), for 260/230 and 260/280 ratios evaluation.

### Genotyping with nuclear and chloroplast SSR, and SNP markers

The 79 accessions were genotyped with 12 nuclear SSR (nSSR) markers. These markers are listed in Table S2 and encompass the nine used for grapevine identification internationally (VVS2, VVMD5, VVMD7, VVMD25, VVMD27, VVMD28, VVMD32, VrZAG62, VrZAG79)^17^, plus ISV2 (VMC6e1), ISV4 (VMC6g1) and VMCNG4b9^18^. The nSSR profiles were obtained using fluorescent primers and an ABI3130xl genetic analyser (Applied Biosystems, Foster City, CA). SSR allele calling was performed with GeneMapper software version 5.0, with a home-made bin set produced with reference varieties. Identifications were made by comparing the obtained genetic profiles with the CREA Viticulture and Enology molecular database, literature information and the *Vitis* International Variety Catalogue (*V*IVC, http://www.vivc.de).

Chlorotypes were determined with eight chloroplast SSR markers out of the nine proposed^19^. Two multiplex PCR were organized using fluorescent primers and SSR allele calling was as described for nSSRs.

All accessions were genotyped using the Infinium® II Vitis18k SNP array, which includes 18,071 SNPs, (GrapeReSeq Consortium, Illumina) following the Infinium® HD Assay Ultra protocol (Illumina Inc., San Diego, CA) and scanned using an Illumina HiScan.

### Data elaboration and parentage relationships

A search for compatible trios (parents and offspring) and duos (parent-offspring) was made based on 9 to 12 nSSRs in the CREA Viticulture and Enology database with Cervus 3.0^20^ and GenAlEx 6.5 software^21^, and in the *V*IVC with the “Relationships based on nine microsatellites” tool. The varieties proving to be members of trios or duos were included in the sample set. These varieties are in italics in Table S1.

SNP data quality was evaluated using GenomeStudio Genotyping Module v2.0 of ILLUMINA. The 18,071 SNPs were selected with three criteria, minimum allele frequency (MAF) higher than 0.05, no calls (NC) lower than 5% and call quality values (p50GC) higher than 0.54. Accordingly, 11,929 SNPs were retained for subsequent analyses. PLINK Input Report Plug-in v2.1.4 for Genome Studio Genotyping module was used to convert genetic data into ‘map’ and ‘ped’ files for analysis with PLINK v1.07 software^22^ (http://pngu.mgh.harvard.edu/purcell/plink). Parent-Offspring (PO) relationships were inferred analyzing four PLINK parameters: the probability of sharing no identical by descent (IBD) allele per locus (Z0), the probability of sharing both IBD alleles per locus (Z2), the probability of sharing one IBD allele per locus (Z1), the PI-HAT (Z2 + 0.5 * Z1). Reference values for PO are Z0 = 0, Z1 = 1, Z2 = 0, PI-HAT = 0.5.

ASSIsT (Automatic SNP ScorIng Tool) software^23^ v. 1.02 was applied for additional SNPs pruning and improved clustering classification of SNPs previously selected using GenomeStudio tools. The aim was to better evaluate pedigree results on putative PO related cultivars conflicting with previous results reported in the literature. ASSIsT default parameters were applied; no SNP map position was given, because ASSIsT does not work with random or unmapped SNPs. The pedigree file was given with mother and father information missing, also for the pedigrees used as references. After pruning, the selected SNPs were analyzed again on a larger set of 192 genotypes to obtain a more consistent SNP classification into the four classes elaborated by ASSIsT: Robust, OneHomozygRare_HWE, OneHomozygRare_notHWE, DistortedAndUnexpSegreg. The more restricted set of higher-quality SNPs was used to recalculate Mendelian inconsistencies on putative PO-related cultivars.

Full-sib relationships were inferred using Colony software version 2.0.6.5 (July 30, 2018), freely available at https://www.zsl.org/science/research-projects/software. Empirical data input consisted of both nSSR and SNP markers. The Parent-Parent-Offspring trios inferred with PLINK and GenomeStudio were used to define the main structure of the family into Colony. The following main settings were applied: markers error rate 0.00001, no sibship prior indicator, one medium run, FL (full likelihood) analysis method and medium precision in calculating FL. Only full-sib dyads with probability equal to 1 were retained. Colony reconstructs the possible genotypes of the parents absent in the dataset and complementing the full-sibs found; these genotypes were used to evaluate additional PO relationships suggested by the software.

MEGA X software version 10.0.5^24^ was used to produce an unrooted dendrogram of genetic similarity using the first-round 11,929 selected SNP markers. Pairwise genetic distances were computed using the Kimura 2-parameter method. Missing data were removed for each sequence pair (pairwise deletion option). The dendrogram was constructed using the Unweighted Pair-Group Arithmetic Average Method (UPGMA). A bootstrap test of 500 replicates was used to define the percentage of replicate trees in which the associated genotypes clustered together; these values were shown next to the branches. Only branches with bootstrap values higher than 75 were taken into consideration.

## Results

The pedigree relationships of FVG grapevine varieties and others from neighboring countries were investigated by a well-established low-throughput SSR approach and a high-throughput genotyping system based on an SNP chip array, the *Vitis* 18k SNP.

SSR genotypes and ampelographic features of most varieties of interest for FVG are available on-line in the Italian *Vitis* database (http://www.vitisdb.it/). *V*IVC prime names and variety numbers for FVG varieties are reported in Table S1. If the prime name differs considerably, it is added in brackets in Table S2, which reports chlorotypes and nSSR profiles of all studied varieties.

Filtering reduced the initial 18,071 SNPs available in the chip of ILLUMINA to 11,929.

All varieties were univocally identifiable with the 14 SNP set selected by^25^; these profiles are provided in Table S3. The SNP complete profiles are in Table S4.

Taking the ‘Heunisch’ samples and related synonyms (‘Liseiret’ and ‘Rebula Stara’), 18 out of 11,929 SNPs failed, all others showed the same allelic combination except one, ‘Heunisch Weiss Seedless’ (data not shown). Concerning the two ‘Ribolla Gialla’ samples, one from the Italian Collio and one from the Slovenian Brda, only 5 SNPs were missing, and the others showed the same allelic profile.

**Figure 1:**
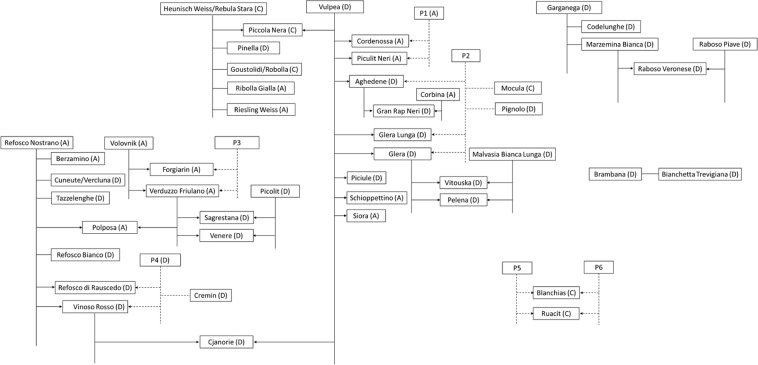
Pedigree reconstruction. In brackets the chlorotype, codified in letters following^19^. Solid lines represent links inferred with molecular data on existing genotypes and vines. Dotted lines indicate presumed full-sib relationships where the complementary or both parents are missing from the dataset.

PLINK, Parent-Parent-Child (P-P-C) error rates and Mendelian inconsistencies of already known kinships were used as reference for discovering new relationships; 12 nSSR data and the chlorotypes were also accounted for. The P-P-C trios found are shown in Table 1 and Fig. 1. One trio involved a well-known and ancient variety of FVG, ‘Piccola Nera’; two pairs of parents were proposed for ‘Piccola Nera’, ‘Pinella’ and ‘Vulpea’ or ‘Heunisch Weiss’ and ‘Vulpea’^8^. These data support ‘Heunisch’ × ‘Vulpea’; moreover, ‘Pinella’ was shown to be half-sib of ‘Piccola Nera’. The other trios regarded varieties detected in the reconnaissance of old vineyards^4,5^ which have been given fantasy names, like ‘Gran Rap Neri’, ‘Pelena’, ‘Polposa’, ‘Sagrestana’ and ‘Venere’. ‘Gran Rap Neri’ derived from ‘Aghedene’ × ‘Corbina’. ‘Sagrestana’ and ‘Venere’ share the same parents, being derived from ‘Picolit’ × ‘Verduzzo Friulano’; ‘Pelena’ is another progeny of ‘Glera’ and ‘Malvasia Bianca Lunga’, like ‘Vitouska’^13^. A possible seventh trio was found: ‘Cjanorie’ could be the progeny of ‘Vulpea’ and ‘Vinoso Rosso’, even if the P-P-C error rate and Mendelian inconsistencies are higher than the references.

**Table 1:** Parents and offspring trios selected combining data from PLINK, GenomeStudio Parent-Parent-Child (P-P-C) error rate and the 12 SSR markers used for identification. MI: Mendelian inconsistencies, computed on trios.

Offspring	First candidate parent	Second candidate parent	PLINK				relation	GenomeStudio	MI
			Z0	Z1	Z2	PI_HAT		P-P-C error rate	
Piccola nera	Heunisch Weiss		0.0518	0.8650	0.0832	0.5157	PO		
		Vulpea	0.0443	0.8865	0.0692	0.5125	PO		
	Heunisch Weiss	Vulpea						0.0071285	0.006157
Gran Rap Neri	Aghedene		0	1	0	0.5	PO		
		Corbina	0	1	0	0.5	PO		
	Aghedene	Corbina						0.0078534	0.005707
Pelena	Malvasia Bianca Lunga		0.043	0.9101	0.0468	0.5019	PO		
		Glera	0.0379	0.8616	0.1005	0.5313	PO		
	Malvasia Bianca Lunga	Glera						0.0077424	0.005456
Polposa	Refosco Nostrano		0.043	0.9428	0.0142	0.4856	PO		
		Verduzzo Friulano	0.0278	0.8795	0.0927	0.5324	PO		
	Refosco Nostrano	Verduzzo Friulano						0.0070226	0.004786
Sagrestana	Picolit		0.0471	0.9529	0	0.4764	PO		
		Verduzzo Friulano	0.0354	0.9236	0.0409	0.5028	PO		
	Picolit	Verduzzo Friulano						0.0081890	0.005626
Venere	Picolit		0.0329	0.9551	0.012	0.4896	PO		
		Verduzzo Friulano	0.0376	0.9624	0	0.4812	PO		
	Picolit	Verduzzo Friulano						0.0072323	0.004783
Cjanorie	Vulpea		0	1	0	0.5	PO		
		Vinoso Rosso	0	1	0	0.5	PO		
	Vulpea	Vinoso Rosso						0.0102984	0.007384
**Reference trios**									
Manzoni Bianco	Pinot		0.0278	0.9537	0.0185	0.4953	PO		
		Riesling Weiss	0.0455	0.8514	0.103	0.5287	PO		
	Pinot	Riesling Weiss						0.0071174	0.005374
Raboso Veronese	Raboso Piave		0	1	0	0.5	PO		
		Marzemina Bianca	0.0442	0.9558	0	0.4779	PO		
	Raboso Piave	Marzemina Bianca						0.0080663	0.006128
Vitouska	Malvasia Bianca Lunga		0.0544	0.8174	0.1281	0.5369	PO		
		Glera	0.0316	0.9076	0.0608	0.5146	PO		
	Malvasia Bianca Lunga	Glera						0.0078600	0.005545

Twenty-three duos were found (Table 2, Fig. 1). Two main recurrent parents stand out among the others: ‘Refosco Nostrano’ and ‘Vulpea’. ‘Refosco Nostrano’ was shown to be first degree linked with varieties related to the Refosco family, like ‘Berzamino’, ‘Tazzelenghe’, ‘Refosco di Rauscedo’ and ‘Refosco Bianco’. Moreover, ‘Refosco Nostrano’ is PO related to the ancient ‘Cuneute’ (also called ‘Vercluna’^26^). ‘Vulpea’ was shown to be PO related to 8 varieties, many of them, i.e. ‘Cordenossa’, ‘Piculit Neri’, ‘Piciule’, ‘Schioppettino’, ‘Siora’ and ‘Aghedene’, are minor or even niche FVG varieties. In addition, a first-degree relationship was detected between ‘Vulpea’ and both ‘Glera’ cultivars, ‘Glera’ and ‘Glera Lunga’. ‘Verduzzo Friulano’ and ‘Forgiarin’ were shown to be PO related to the Slovenian ‘Volovnik’, also known under the synonym ‘Volovna’; molecular data exclude that the two FVG varieties could be the parents of ‘Volovnik’.

**Table 2:** PLINK parameter values and Mendelian inconsistencies (MI) for pairs of parent-offspring (PO) related varieties and pairs with conflicting data in respect to the previously proposed PO kinship. MI are shown also computed on the more restricted group of 9,177 SNPs selected through ASSIsT software and classified using 192 genotypes. DAUS: DistortedAndUnexpSegreg, OHR: OneHomozygRare_HWE and OneHomozygRare_notHWE.

Varieties with supporting data		PLINK 11,929 SNPs					MI computed on 9,177 SNPs selected through ASSIsT					Percentage of total MI reduction based on ASSIsT
		Z0	Z1	Z2	PI_HAT	MI total number	MI total number	MI on Robust SNPs	MI on DAUS SNPs	MI on OHR SNPs	MI (Robust + OHR)	
Brambana	Bianchetta Trevigiana	0.0341	0.9556	0.0103	0.4881	27	13	3	9	1	4	51.9
Garganega	Codelunghe	0.0342	0.9658	0	0.4829	28	19	7	11	1	8	32.1
	Marzemina Bianca	0.0493	0.921	0.0297	0.4902	39	24	9	14	1	10	38.5
Heunisch weiss	Pinella	0.0387	0.9613	0	0.4806	31	19	6	11	2	8	38.7
	Riesling Weiss	0.0529	0.9471	0	0.4736	44	31	5	18	8	13	29.5
	Ribolla Gialla	0.0461	0.9539	0	0.4769	37	27	8	15	4	12	27.0
	Goustolidi	0.0392	0.8549	0.1059	0.5333	31	17	7	9	1	8	45.2
Refosco nostrano	Berzamino	0.0493	0.8742	0.0764	0.5136	39	24	8	14	2	10	38.5
	Cuneute	0.0442	0.8927	0.063	0.5094	35	21	9	11	1	10	40.0
	Refosco Bianco	0.0329	0.9328	0.0344	0.5007	26	15	4	10	1	5	42.3
	Refosco di Rauscedo	0.0316	0.9506	0.0178	0.4931	25	15	6	8	1	7	40.0
	Tazzelenghe	0.0341	0.8834	0.0824	0.5242	27	15	3	12	0	3	44.4
	Vinoso Rosso	0.0202	0.9380	0.0418	0.5108	16	10	4	6	0	4	37.5
Volovnik	Forgiarin	0	1	0	0.5	39	31	12	17	2	14	20.5
	Verduzzo friulano	0	1	0	0.5	39	29	11	17	1	12	25.6
Vulpea	Aghedene	0.0557	0.9443	0	0.4722	45	29	5	19	5	10	35.6
	Cordenossa	0	1	0	0.5	43	28	8	15	5	13	34.9
	Glera Lunga	0.0399	0.9601	0	0.4801	32	9	0	8	1	1	71.9
	Glera	0	1	0	0.5	35	19	8	9	2	10	45.7
	Piciule	0	1	0	0.5	41	25	6	14	5	11	39.0
	Piculit Neri	0	1	0	0.5	41	25	4	18	3	7	39.0
	Schioppettino	0.0548	0.9452	0	0.4726	45	33	7	21	5	12	26.7
	Siora	0.0517	0.9483	0	0.4741	43	26	6	16	4	10	39.5
**Varieties with conflicting data**												
Glera lunga	Glera	0.1226	0.5798	0.2976	0.5875	97	88	44	36	8	52	9.3
Marzemino	Marzemina Bianca	0.4965	0.5035	0	0.2517	404	386	172	135	79	251	4.5
Marzemino	Refosco dal Peduncolo Rosso	0.1301	0.5961	0.2738	0.5718	103	90	39	36	15	54	12.6
Marzemino	Teroldego	0.2667	0.6188	0.1145	0.4239	211	188	77	61	50	127	10.9
**Reference PO**												
Traminer	Pinot	0.0316	0.8279	0.1405	0.5545	25	17	8	7	2	10	32.0
Traminer	Sauvignon	0.0462	0.9538	0	0.4769	37	27	10	14	3	13	27.0

Two pairs of varieties were shown to be full-sib related, ‘Vitouska’ and ‘Pelena’, ‘Sagrestana’ and ‘Venere’. Colony inferred some additional FS relationships, represented by dotted lines in Fig. 1 and involving i) ‘Cordenossa’ and ‘Piculit Neri’, ii) ‘Aghedene’, ‘Glera’ and ‘Glera Lunga’, iii) ‘Forgiarin’ and ‘Verduzzo Friulano’, iv) ‘Refosco di Rauscedo’ and ‘Vinoso Rosso’.

‘Marzemina Bianca’ and ‘Codelunghe’ were shown to be PO related to ‘Garganega’, like ‘Brambana’ to ‘Bianchetta Trevigiana’; moreover, ‘Ruacit’ and ‘Blanchias (Blancjàs)’ could also be FS related (Fig. 1).

**Figure 2:**
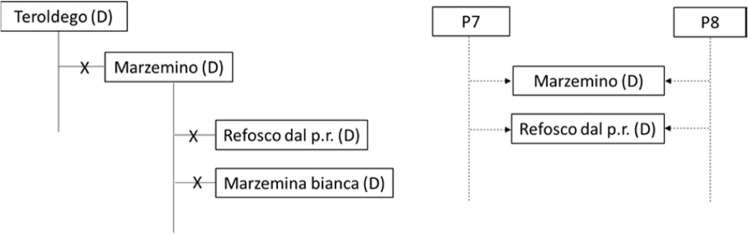
Rejected kinships and new proposal.

Some pedigrees derived from previous SSR studies were not supported by the SNP chip analysis, because PLINK parameters and Mendelian inconsistencies were incompatible with PO relationships compared to the references (Table 2). In more detail, Z1 value was expected to be not less than 0.8279, given the references used, instead, the values found were too low and only between 0.5035 and 0.6188. Regarding Mendelian inconsistencies (MI), PO relationships given as references are 0.0021 for Pinot and Traminer, and 0.0031 for Traminer and Sauvignon Blanc; MI showed to be a little bit higher for Riesling Weiss and Heunisch (0.0037), a pair of varieties already assumed to be PO related by the literature data (*V*IVC). MI lower than or very close to the highest value for the reference pairs (0.0037) were computed for the pairs of varieties considered to be PO related; instead, MI 2 to 9 times higher than the highest value of reference were computed for pairs excluded as being PO related. Specifically, our data reject the PO relationships for ‘Marzemino’ and ‘Teroldego’ and for ‘Marzemino’ and ‘Refosco dal Peduncolo Rosso’ supported by other studies^8,27^. Furthermore, SNP data do not support the PO relationship for ‘Marzemino’ and ‘Marzemina Bianca’^28^ nor for ‘Glera Lunga’ and ‘Glera’^8^. Instead, Colony output supported the hypothesis that ‘Marzemino’ and ‘Refosco dal Peduncolo Rosso’ could be FS related (Fig. 2).

ASSIsT software was applied to the set of 11,929 SNPs previously selected using GenomeStudio tools: 9,177 SNPs (76.93%) were retained and more consistently classified using a larger set of 192 genotypes. The 9,177 SNPs were used for Mendelian inconsistencies (MI) computation. The obtained results are reported in Table 2. ASSIsT-mediated SNP-pruning decreased MI absolute number from 4.5% for Marzemino/Marzemina Bianca pair to 71.9% for Vulpea/Glera Lunga pair. The lowest MI improvement was for varieties with conflicting data, going from 4.5 to 12.6%; instead, the greatest improvement was for all other pairs, showing values from 20.5 to 71.9%. The reference duos showed MI total number of 17 and 27; the varieties with supporting data had values from 9 to 33, among them Riesling Weiss with 31 total MI. Varieties with conflicting data showed higher total MI, from 88 to 386, than varieties with supporting data, which is at least 2.7 times higher than supported PO relationships.

MI were also split in three classes: Robust, DistortedAndUnexpSegreg, and OneHomozygousRare (encompassing both OneHomozygousRare_HWE and OneHomozygousRare_not HWE) (Table 2). Given that MI based on DistortedAndUnexpSegreg can be ascribed to AB × AO marker in germplasm population, Robust and OneHomozygousRare based MI were the strongest reference values for PO relationships evaluation and were merged into one class in the penultimate column of Table 2. By comparing the MI in the merged class, the reference duos showed values of 10 and 13; the varieties with supporting data values from 1 (‘Glera Lunga’) to 14 (‘Forgiarin’); the varieties with conflicting data values from 52 for ‘Glera’/‘Glera Lunga’ pair to 251 for ‘Marzemino’/‘Marzemina Bianca’. The merged class MI was therefore at least 3.7 times higher for varieties with conflicting data than for those with supporting data. In conclusion, ASSIsT-mediated MI values better supported all the PO relationships based on PLINK parameters and still invalidated varieties with conflicting data compared to previous results reported in the literature.

Finally, Colony inferred the nSSR genotype of the presumed common parent for FS related ‘Aghedene’, ‘Glera’ and ‘Glera Lunga’ varieties (P2 in Fig. 1 and Table S5). This genetic profile was checked in combination with ‘Vulpea’ for all three trios, and no mismatching alleles were found. The same genotype was checked for possible PO relationships with the other varieties analyzed in this study using GenAlEx and two additional perfect matches were found, one with ‘Mocula’ and the other with ‘Pignolo’ (Fig. 1).

**Figure 3:**
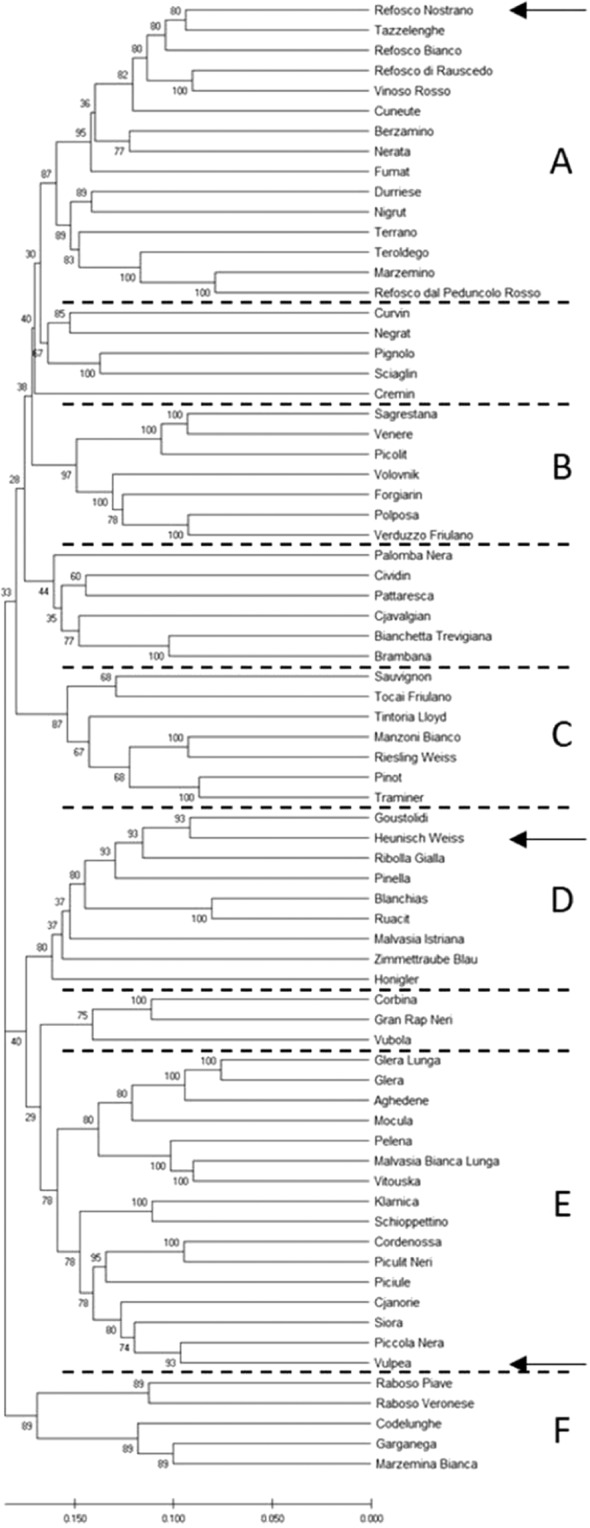
Unrooted optimal phylogenetic tree produced via UPGMA method. The evolutionary distances were computed using the Kimura 2-parameter method with bootstrap test (500 replicates). Arrows point to the three most prominent founder varieties.

The dendrogram of genetic similarity produced via UPGMA divided the studied genotypes into different clusters, six of them showing significant bootstrap values (Fig. 3). As can be seen, genotypes clustered together according to the parental tree shown in Fig. 1. The significant clusters, labeled with alphabetical letters and dotted lines, varied in size from five to 16 varieties. A, the family group of ‘Refosco Nostrano’, 15 varieties, seven of them strictly related; this group also encompassed Teroldego, Marzemino and Refosco dal Peduncolo Rosso. B, the family group of ‘Volovnik’, ‘Verduzzo Friulano’ and ‘Picolit’, showing seven interrelated cultivars; C, the family group of ‘Traminer’, with seven cultivars, five of them interrelated; D, the group of ‘Heunisch’, with nine cultivars, three of them being ‘Heunisch’ offspring; E, the family group of ‘Vulpea’, with 16 cultivars, ten of them being ‘Vulpea’ offspring; F, a very clearly distinct group including five cultivars related to ‘Garganega’ and ‘Raboso Piave’ as shown on the right in Fig. 1.

## Discussion

Parentage analysis was undertaken in a two-step approach commonly used when searching for pedigrees in grapevine^29–31^. Initially, the FVG varieties genotyped with 12 nSSRs were scored for possible PO relationships using the *V*IVC and CREA Viticulture and Enology molecular databases, each one encompassing around 3900 unique genetic profiles. The refined dataset was then used for the analysis with the 18k SNP chip of ILLUMINA.

After filtering, 66% of the total SNP markers available in the chip were used for subsequent analyses.

Our data agree with^25^ that just the 14 SNPs they selected from ILLUMINA chip for identification purposes were enough to also identify all the cultivars analyzed in this study. This is interesting because SNP are qualitative markers and, unlike SSRs, do not need conversion to be comparable with the data produced in different labs using reference varieties. If further confirmed, the shared application of this set of 14 SNP markers would open the possibility of reducing the cost of this kind of analysis and would make genotyping also only one sample at a time economically sustainable.

The present FVG grapevine varietal assortment showed no cultivars derived from self-pollination. As a rule, a low number of generations was detected and some ancient varieties can be regarded as “founders”, like ‘Vulpea’ and ‘Refosco Nostrano’. Our findings agree with what was pointed out at a broader level of pedigree complexity^8^.

Surprisingly, ‘Vulpea’, which was classified in the *V*IVC as a Romanian variety, is present in FVG, where it counted among the ancient local varieties as anonymous or even misnomered vines. For example, genotyping of old vines with 12 nSSRs revealed ‘Vulpea’ under wrong designations like ‘Codelunghe’ and ‘Piccola Nera’^3^. Moreover, again by means of genotyping, we recognized ‘Vulpea’ in the nearby Veneto region, under the name of ‘Quaiara’ in Verona province and as ‘Rossetta’ or ‘Sciavetta’ or ‘Doretta’ on the Euganean Hills, near Padua, where it has been cultivated with some success to produce a popular rosé table wine. Information on ‘Quaiara’ and ‘Rossetta/Sciavetta/Doretta’ is available in^32,33^. A comparison of ‘Vulpea’ ampelographic description published in the European *Vitis* Database (http://www.eu-vitis.de, accession number ROM051-272) with ‘Quaiara’ and ‘Rossetta/Sciavetta/Doretta’ accessions grown at CREA-Viticulture and Enology repository in Susegana (TV) evidenced matching morphology (Severina Cancellier, personal communication). In the 19^th^ century, ‘Vulpea’ existed in nearly every Austrian and northeast Slovenian vineyard^34,35^ and in Siebenbürgen/Romania^36^. The Austrian Helbling provided the first accurate description in 1777 under the name ‘Schwarzer Abendroth’^37^. According to ampelographers of the 18^th^ and 19^th^ centuries the late maturating ‘Vulpea’ produced bunches with green, red and blue berries. The wine was of low quality, had a light red color and sour taste, however the cultivar was appreciated for its heavy crop. Similar to ‘Heunisch Weiss’, it was recommended for “eradication”^35^. ‘Vulpea’ is grown successfully in the warmer Italy^34^, thus indicating that ‘Vulpea’ spread from neighboring countries to northeast Italy, probably since ancient times.

The parents of ‘Vulpea’ are the Croatian ‘Bratkovina Crna’ and Hungarian ‘Gyoengy Feher’^8^. Therefore, according to present knowledge, all FVG cultivars first-degree related to ‘Vulpea’ should be offspring of this variety.

The discovery of Vulpea’s high impact on the FVG variety assortment and the involvement of the Croatian ‘Bratkovina Crna’ and Hungarian ‘Gyoengy Feher’ in its ancestry pointed more to Austria as the country of origin than to Romania or Moldavia. In the latter ‘Vulpea’, known as ‘Ciorcuta Rosie’, was also considered autochthonous.

‘Refosco Nostrano’ showed strong links with all other Refosco varieties, except for ‘Refosco dal Peduncolo Rosso’. ‘Refosco Nostrano’ was pivotal for some niche varieties, to our knowledge, only grown in a specific and restricted area of FVG: ‘Berzamino’, ‘Tazzelenghe’, ‘Refosco di Rauscedo’ and ‘Refosco Bianco’. In FVG all varieties were reproduced locally before the arrival of phylloxera. Afterwards, grafting was performed mostly in large nurseries where only some varieties, mainly the most productive, were propagated, endangering biodiversity conservation. In the small towns of Faedis and Nimis, far distant from the main nursery in Cividale del Friuli, grafting continued to be done on-site, saving some autochthonous varieties from extinction. In detail, ‘Berzamino’, ‘Curvin’ and ‘Tazzelenghe’ (locally called ‘Refosco dal Botton’) were found in Nimis, while ‘Refosco Nostrano’, ‘Vinoso Rosso’, ‘Piculit Neri’ and ‘Siora’ in Faedis. All these varieties were propagated for their high yield. Historically, all Refosco varieties (‘Refosco dal Peduncolo Rosso’, ‘Terrano’, ‘Refosco Nostrano’, ‘Berzamino’ and ‘Tazzelenghe’) were widely distributed in FVG, from the hills to the sea^38^; among these varieties ‘Refosco dal Peduncolo Rosso’ was widely cultivated, while the others were only grown locally. Instead, ‘Refošk’ (synonym ‘Teran’), corresponding to the FVG ‘Terrano’, is the most cultivated in Kras (Carst) and Istria^7^.

‘Cjanorie’ appears to be a bridge between ‘Vulpea’ and ‘Refosco Nostrano’. ‘Cjanorie’ is PO related to ‘Vulpea’ and ‘Vinoso Rosso’. Moreover, no pair of varieties in PO with ‘Refosco Nostrano’ can be the parents of this ancient variety and Colony indicates an FS relationship between ‘Refosco di Rauscedo’ and ‘Vinoso Rosso’. Consequently, the only possible kinship for ‘Cjanorie’ seems to be that it is the progeny of ‘Vulpea’ and ‘Vinoso Rosso’.

‘Ribolla Gialla’ cultivar of the Italian Collio corresponds to the ‘Rebula’ grown in Goriška Brda, the Slovenian Collio, as confirmed with genotyping^39^. The same authors analyzed a so-called ‘Rebula Stara’, meaning “old Rebula”. This variety was shown to be different from ‘Ribolla Gialla’ and possibly PO related, according to 11 SSRs. It was a surprise to discover that ‘Rebula Stara’ corresponded to ‘Heunisch Weiss’ after molecular profile comparison with available databases. ‘Heunisch Weiss’ is a variety of paramount importance for the evolution of grapevine varietal assortment. The pair ‘Pinot’ and ‘Heunisch Weiss’ originated a dozen French varieties^40^, among them ‘Chardonnay’ and ‘Gamay’; in addition, ‘Heunisch Weiss’ could be the parent of more than a hundred varieties, even outside France, such as in Germany, Greece, Hungary and Romania^8^. Three years after the work by^39^, the hypothesis of a PO relationship between ‘Ribolla Gialla’ and ‘Heunisch Weiss’ using 20 SSRs was again supported by^8^. However, this parentage was ruled out by^9^, because five mismatching markers were found using a deep genotyping with 58 SSRs and these incompatibilities were considered too many. These authors did not take into consideration another aspect that can explain the mismatches found. In ancient varieties propagated vegetatively for centuries, molecular differences can accumulate as in a biological clock. For example, a wide clonal diversity was found in ‘Savagnin’^41^, a very ancient variety at least 900 years old^42^, using only 30 SSRs: 49 plants were analyzed^41^ and only 12 showed the same genotype, i.e. 24.5%. In conclusion, if the genotyping is restricted to only one plant, such differences cannot be evidenced, and molecular information could lead to erroneous conclusions. For this reason, six accessions of ‘Heunisch’, coming from three countries were compared in this work, some of them being phenotypic variants^16^, and two ‘Ribolla Gialla’ accessions. SNP data strongly support a first-degree relationship between ‘Heunisch Weiss’ and ‘Ribolla Gialla’.

‘Robola’ sounds very similar to ‘Rebula’ and ‘Ribolla’. ‘Robola’ is also homonym for different varieties grown in Greece. A ‘Robolla’ sample coming from Greece and corresponding to ‘Goustolidi’ (*V*IVC variety number 5000) was added to our varietal list, to understand if there was some link among these varieties. All available genotyping data exclude that ‘Ribolla Gialla’ is currently grown in Greece as any one of the genotyped ‘Robola’ cultivars^9,43^. Moreover, ‘Robola/Goustolidi’ was shown to be another Greek variety PO related to ‘Heunisch Weiss’, supporting a previous finding^8^.

Other local Italian varieties, such as ‘Pinella’ (Veneto) and ‘Piccola Nera’ (FVG) showed possible PO relationship with ‘Heunisch Weiss’ according to 20 SSR markers^8^; SNP data confirmed those results. The Slovenian ‘Volovnik’ was shown to be first degree related to two FVG varieties, ‘Forgiarin’ and ‘Verduzzo Friulano’, and is possibly one of their parents, while no relationship was found for ‘Klarnica’.

‘Mocula’ and ‘Pignolo’ could be half-sib of ‘Aghedene’, ‘Glera’ and ‘Glera Lunga’. These additional pedigree relationships reinforce the hypothesis that the P2 vine really existed in the past. However, recovery of the P2 genotype is necessary to confirm these assumptions.

The dendrogram constructed by UPGMA is a fairly faithful representation of already known pedigree relationships and the new ones found in this research, in agreement with the relatedness proposed and summarized in Fig. 1. Most of the offspring were directly aligned to the fourteen putative parents. The dendrogram also represents a source of information to further investigate the traditional varieties genetic backgrounds or relationships.

The cluster analysis clearly separated the three most prominent founder varieties of this study ‘Vulpea’, ‘Refosco Nostrano’ and ‘Heunisch’, pointing to their distinct geographic and genetic backgrounds. These three varieties created large family groups. The fairly stringent grouping leading to the clear separation observed between variety families can be due to the conscious choice of additional cultivars (Table S2), which turned out to be related to traditional varieties in FVG. However, placement in the dendrogram can be critical in the case of “bridge” varieties, like ‘Piccola Nera’ (‘Heunisch’ × ‘Vulpea’) and Cjanorie (‘Vulpea’ × ‘Refosco Nostrano’), both grouped with ‘Vulpea’, or for varieties with still unclear pedigree, like ‘Riesling Weiss’, grouped with ‘Traminer’ even if PO related to ‘Heunisch’. In this last case the reason may reside in the 2^nd^ parent being very close to Traminer and the wild grape (Erika Maul, personal communication) and on the “attraction” by ‘Manzoni Bianco’ (‘Pinot’ × ‘Riesling Weiss’).

## Conclusions

Genotyping studies provide a more stringent and sometimes new perspective on the origin, spread and relatedness of grapevine cultivars. Research on pedigrees highlighted that outcrossing is the main strategy for the birth of new varieties, whilst very few cultivars derive from selfing; it has now become recognized that grape flowers are subjected not only to cleistogamy, but also to pollination by wind or insects. Shedding light on grapevine pedigree relationships helps in understanding the germplasm assortment evolution and cultivar history. Some varieties of FVG are shared with neighboring areas, in and outside Italy, like ‘Glera’, ‘Glera Lunga’, ‘Ribolla Gialla’ and ‘Piccola Nera’, others are specific to FVG. Even their history intertwines with local and foreign varieties. Surprisingly, ‘Vulpea’ was shown not only to be spread in FVG and Veneto since ancient times, but it was also recognized as a recurrent parent of at least ten FVG cultivars, both ancient and well-known, like ‘Glera’, as well as recent or neglected. Many ‘Refosco’ varieties are PO related to ‘Refosco Nostrano’, showing that Refosco’s represent a real family; ‘Refosco dal Peduncolo Rosso’ does not strictly belong to this family and is likely full-sib related to ‘Marzemino’. ‘Ribolla Gialla’ was shown to be PO related to ‘Heunisch Weiss’, therefore being another of the numerous offspring derived from this prolific variety.

In conclusion, combining molecular markers and historical references was shown to be a high-performance strategy to retrace and adjust the history of cultivars.

## Supplementary information

Supplementary information

Supplementary information 2
