# Author Correction: Unraveling the genetic origin of ‘Glera’, ‘Ribolla Gialla’ and other autochthonous grapevine varieties from Friuli Venezia Giulia (northeastern Italy)

**DOI:** 10.1038/s41598-021-90277-5

**Published:** 2021-05-12

**Authors:** Manna Crespan, Daniele Migliaro, Simone Larger, Massimo Pindo, Carlo Petrussi, Marco Stocco, Denis Rusjan, Paolo Sivilotti, Riccardo Velasco, Erika Maul

**Affiliations:** 1 CREA Research Centre of Viticulture and Enology, Conegliano, Treviso, Italy; 2 Unit of Computational Biology, Research and Innovation Centre, Fondazione Edmund Mach, San Michele all’Adige, Trento, Italy; 3 Viticulturist, Cividale del Friuli, Udine Italy; 4 ERSA, Agenzia Regionale Per Lo Sviluppo Rurale, Pozzuolo del Friuli, Udine Italy; 5 Department of Agronomy, Biotechnical Faculty, University of Ljubljana, Ljubljana, Slovenia; 6 Department of Agricultural, Food, Environmental and Animal Sciences, University of Udine, Udine, Italy; 7 JKI - Institute for Grapevine Breeding Geilweilerhof, Siebeldingen, Germany

Correction to: *Scientific Reports* 10.1038/s41598-020-64061-w, published online 29 April 2020

The Supplementary Tables 3S and 4S published with this Article contain errors, where data for the SNP profiles was coded incorrectly.

The correct Supplementary Information tables are linked to this correction notice.

## Supplementary Information

Supplementary Table 3S.

Supplementary Table 4S.

