# Supplementary material for: Unraveling the genetic origin of ‘Glera’, ‘Ribolla Gialla’ and other autochthonous grapevine varieties from Friuli Venezia Giulia (northeastern Italy)
Source: Sci Rep. 2020 Apr 29;10:7206. doi: 10.1038/s41598-020-64061-w (PMC7190720; doi:10.1038/s41598-020-64061-w)
Supplement: Supplementary file 1 — Supplementary information [file 41598_2020_64061_MOESM1_ESM.pdf]

Unraveling the genetic origin of ‘Glera’, ‘Ribolla Gialla’ and other autochthonous grapevine varieties from Friuli Venezia Giulia (northeastern Italy)

Manna Crespan, Daniele Migliaro, Simone Larger, Massimo Pindo, Carlo Petrussi, Marco Stocco, Denis Rusjan, Paolo Sivilotti, Riccardo Velasco, Erika Maul

Table 1S - List of the analysed samples. Non-FVG varieties are in italics; additional varieties with known pedigree relationships used as reference for computation are in bold.

ID	*V*IVC prime name	Variety/accession name	Repository (Institute code)	Country of origin	Variety Number *V*IVC
1	AGHEDENE B	Aghedene	CREA-Viticulture and Enology, Susegana (TV) (ITA388)	ITALY	23351
2	BERZAMINO N	Berzamino	CREA-Viticulture and Enology, Susegana (TV) (ITA388)	ITALY	24422
3	BIANCHETTA TREVIGIANA B	*Bianchetta trevigiana*	CREA-Viticulture and Enology, Susegana (TV) (ITA388)	ITALY	1329
4	BLANCJAS B	Blanchias*	CREA-Viticulture and Enology, Susegana (TV) (ITA388)	ITALY	24448
5	BRAMBANA	Brambana	CREA-Viticulture and Enology, Susegana (TV) (ITA388)	ITALY	1663
6	CIVIDIN BIANCO B	Cividin	CREA-Viticulture and Enology, Susegana (TV) (ITA388)	ITALY	2690
7	CIANORIE N	Cjanorie	CREA-Viticulture and Enology, Susegana (TV) (ITA388)	ITALY	2644
8	CJAVALGJAN N	Cjavalgian	ERSA, Maniago (Pordenone), Italy	ITALY	23349
9	CODELUNGE N	Codelunghe	CREA-Viticulture and Enology, Susegana (TV) (ITA388)	ITALY	2744
10	CORBINONA N	Corbina	CREA-Viticulture and Enology, Susegana (TV) (ITA388)	ITALY	24567
11	CORDENOSSA N	Cordenossa	CREA-Viticulture and Enology, Susegana (TV) (ITA388)	ITALY	23018
12	CREMIN N	Cremin*	CREA-Viticulture and Enology, Susegana (TV) (ITA388)	ITALY	24451
13	CUNEUTE N	Cuneute	CREA-Viticulture and Enology, Susegana (TV) (ITA388)	ITALY	3293
14	CURVIN N	Curvin	ERSA, Maniago (Pordenone), Italy	ITALY	24452
15	DURIESE B	Durriese	ERSA, Maniago (Pordenone), Italy	ITALY	24530
16	FORZARIN N	Forgiarin	CREA-Viticulture and Enology, Susegana (TV) (ITA388)	ITALY	4202
17	FUMAT N	Fumat	CREA-Viticulture and Enology, Susegana (TV) (ITA388)	ITALY	24418
18	GARGANEGA B	*Garganega*	CREA-Viticulture and Enology, Susegana (TV) (ITA388)	ITALY	4419
19	GLERA B	Glera	CREA-Viticulture and Enology, Susegana (TV) (ITA388)	ITALY	9741
20	BELA DINKA B	Glera lunga	CREA-Viticulture and Enology, Susegana (TV) (ITA388)	SERBIA	16848
21	GRAN RAP NERI N	Gran rap neri*	CREA-Viticulture and Enology, Susegana (TV) (ITA388)	ITALY	24455
22	HEUNISCH DREIFARBIG RS	*Heunisch dreifarbig*	JKI, Institute for Grapevine Breeding Geilweilerhof (DEU098)	AUSTRIA	24544
23	HEUNISCH ROTGESTREIFT B	*Heunisch rotgestreift*	JKI, Institute for Grapevine Breeding Geilweilerhof (DEU098)	AUSTRIA	5373
24	HEUNISCH WEISS B	*Heunisch weiss*	JKI, Institute for Grapevine Breeding Geilweilerhof (DEU098)		5374
25	HEUNISCH WEISS SEEDLESS B	*Heunisch weiss seedless*	JKI, Institute for Grapevine Breeding Geilweilerhof (DEU098)		699
26	HONIGLER B	*Honigler*	CREA-Viticulture and Enology, Susegana (TV) (ITA388)	HUNGARY	5417
27	KLARNICA B	*Klarnica*	STS - Vrhpolje (Vipava) (SVN018)	SLOVENIA	40687
28	HEUNISCH WEISS B	*Liseiret*	CREA-Viticulture and Enology, Susegana (TV) (ITA388)		5374
29	MALVASIA BIANCA LUNGA B	**Malvasia bianca lunga**	CREA-Viticulture and Enology, Susegana (TV) (ITA388)	ITALY	7262
30	MALVASIA ISTRIANA B	Malvasia istriana	CREA-Viticulture and Enology, Susegana (TV) (ITA388)	CROATIA	7269
31	MANZONI BIANCO B	**Manzoni bianco**	CREA-Viticulture and Enology, Susegana (TV) (ITA388)	ITALY	7360
32	MARZEMINA BIANCA B	**Marzemina bianca**	CREA-Viticulture and Enology, Susegana (TV) (ITA388)	ITALY	16944
33	MARZEMINO N	*Marzemino*	CREA-Viticulture and Enology, Susegana (TV) (ITA388)	ITALY	7463
34	MOCULA B	Mocula*	CREA-Viticulture and Enology, Susegana (TV) (ITA388)	ITALY	24456
35	NEGRAT N	Negrat	ERSA, Maniago (Pordenone), Italy	ITALY	8450
36	NERATA N	Nerata*	ERSA, Maniago (Pordenone), Italy	ITALY	24457
37	NIGRUT N	Nigrut*	CREA-Viticulture and Enology, Susegana (TV) (ITA388)	ITALY	24458
38	PALOMBA N	Palomba nera	ERSA, Maniago (Pordenone), Italy	ITALY	20370
39	PELENA B	Pelena*	ERSA, Maniago (Pordenone), Italy	ITALY	24459
40	PICCOLA NERA RS	Piccola nera	CREA-Viticulture and Enology, Susegana (TV) (ITA388)	ITALY	9235
41	PICULE N	Piciule*	CREA-Viticulture and Enology, Susegana (TV) (ITA388)	ITALY	24460
42	PICOLIT B	Picolit	Vivai Cooperativi di Rauscedo (Pordenone), Italy	ITALY	9236
43	PICOLIT NERO N	Piculit neri	CREA-Viticulture and Enology, Susegana (TV) (ITA388)	ITALY	9238
44	PIGNOLO N	Pignolo	CREA-Viticulture and Enology, Susegana (TV) (ITA388)	ITALY	9255
45	PINELLA B	Pinella	CREA-Viticulture and Enology, Susegana (TV) (ITA388)	ITALY	9266

46	PINOT NOIR N	**Pinot noir**	CREA-Viticulture and Enology, Susegana (TV) (ITA388)	FRANCE	9279
47	POLPOSA B	Polposa*	CREA-Viticulture and Enology, Susegana (TV) (ITA388)	ITALY	24462
48	RABOSO PIAVE N	**Raboso Piave**	CREA-Viticulture and Enology, Susegana (TV) (ITA388)	ITALY	9864
49	RABOSO VERONESE N	**Raboso veronese**	CREA-Viticulture and Enology, Susegana (TV) (ITA388)	ITALY	9865
50	RIBOLLA GIALLA B	Rebula	STS - Vrhpolje (Vipava) (SVN018)	ITALY	10054
51	HEUNISCH WEISS B	*Rebula stara*	STS - Vrhpolje (Vipava) (SVN018)		5374
52	REFOSCO BIANCO B	Refosco bianco	CREA-Viticulture and Enology, Susegana (TV) (ITA388)	ITALY	24463
53	REFOSCO NOSTRANO N	Refosco nostrano	CREA-Viticulture and Enology, Susegana (TV) (ITA388)	ITALY	9989
54	REFOSCO DAL PEDUNCOLO ROSSO N	Refosco dal peduncolo rosso	CREA-Viticulture and Enology, Susegana (TV) (ITA388)	ITALY	9987
55	REFOSCO GENTILE N	Refosco di Rauscedo*	ERSA, Maniago (Pordenone), Italy	ITALY	24536
56	RIBOLLA GIALLA B	Ribolla gialla	CREA-Viticulture and Enology, Susegana (TV) (ITA388)	ITALY	10054
57	RIESLING WEISS B	**Riesling**	CREA-Viticulture and Enology, Susegana (TV) (ITA388)	GERMANY	10077
58	GOUSTOLIDI B	*Robolla*	Vivai Cooperativi di Rauscedo (Pordenone), Italy	GREECE	5000
59	RUACIT B	Ruacit	CREA-Viticulture and Enology, Susegana (TV) (ITA388)	ITALY	24464
60	SAGRESTANA B	Sagrestana*	CREA-Viticulture and Enology, Susegana (TV) (ITA388)	ITALY	23350
61	SAUVIGNON BLANC B	**Sauvignon**	CREA-Viticulture and Enology, Susegana (TV) (ITA388)	FRANCE	10790
62	ZIMMETTRAUBE BLAU N	Sbulzina*	CREA-Viticulture and Enology, Susegana (TV) (ITA388)	SLOVENIA	24493
63	SCHIOPPETTINO N	Schioppettino	CREA-Viticulture and Enology, Susegana (TV) (ITA388)	ITALY	10830
64	SCIAGLIN B	Sciaglin	CREA-Viticulture and Enology, Susegana (TV) (ITA388)	ITALY	17607
65	SIORA N	Siora*	CREA-Viticulture and Enology, Susegana (TV) (ITA388)	ITALY	24467
66	TAZZELENGHE N	Tazzelenghe	CREA-Viticulture and Enology, Susegana (TV) (ITA388)	ITALY	12291
67	TEROLDEGO N	*Teroldego*	CREA-Viticulture and Enology, Susegana (TV) (ITA388)	ITALY	12371
68	TERRANO N	Terrano	CREA-Viticulture and Enology, Susegana (TV) (ITA388)	ITALY	12374
69	TINTORIA LLOYD N	Tintoria Lloyd	CREA-Viticulture and Enology, Susegana (TV) (ITA388)	ITALY	24446
70	FRIULANO B	Tocai friulano	CREA-Viticulture and Enology, Susegana (TV) (ITA388)	ITALY	12543
71	GEWUERZTRAMINER RG	**Traminer**	CREA-Viticulture and Enology, Susegana (TV) (ITA388)		12609
72	UCELUT B	Ucelut	CREA-Viticulture and Enology, Susegana (TV) (ITA388)	ITALY	12753
73	VENERE B	Venere*	CREA-Viticulture and Enology, Susegana (TV) (ITA388)	ITALY	24468
74	VERDUZZO FRIULANO B	Verduzzo friulano	CREA-Viticulture and Enology, Susegana (TV) (ITA388)	ITALY	12976
75	VINOSO ROSSO N	Vinoso rosso	CREA-Viticulture and Enology, Susegana (TV) (ITA388)	ITALY	24469
76	VITOVSKA B	Vitouska	CREA-Viticulture and Enology, Susegana (TV) (ITA388)	SLOVENIA	24412
77	VELA PERGOLA B	*Volovnik*	STS - Vrhpolje (Vipava) (SVN018)	CROATIA	22299
78	VUBOLA B	Vubola*	ERSA, Maniago (Pordenone), Italy	ITALY	24470
79	VULPEA N	*Vulpea*	CREA-Viticulture and Enology, Susegana (TV) (ITA388)	AUSTRIA	13186

*fantasy name made-up by the collector

Table 2S - Chlorotypes and nSSR profiles of the analyzed varieties. The original varietal names reported in Table 1S are here in brackets, when a different prime name is available. Chlorotypes are codified in letters following [19]; allele lenghts are in bp. Non-FVG varieties recognized as members of trios or duos are listed as additional varieties.

**Varieties of Friuli Venezia Giulia**	**Chlorotype**	**VVS2**		**VVMD27**		**VVMD7**		**ISV2 (VMC6E1)**		**VrZAG62**		**VVMD5**		**VMCNG4B9**		**VrZAG79**		**ISV4 (VMC6G1)**		**VVMD28**		**VVMD25**		**VVMD32**	
Aghedene	D	135	155	185	194	239	247	141	151	193	195	226	246	150	176	242	248	177	187	247	251	247	259	257	273
Berzamino	A	151	155	181	185	257	263	165	169	193	195	232	240	162	172	250	258	169	177	249	271	261	267	253	263
Blanchias	C	133	153	181	189	247	249	141	143	199	203	226	234	138	176	242	250	177	177	239	249	245	245	251	263
Brambana	D	135	151	185	189	247	257	151	161	195	203	232	236	158	158	242	250	169	191	261	271	253	259	273	273
Cividin	D	141	143	181	185	247	247	141	143	195	201	236	238	150	162	242	258	169	191	247	261	259	261	241	273
Cjanorie	D	133	155	185	194	239	249	141	151	187	199	226	228	158	162	242	248	169	197	237	251	243	261	265	273
Cjavalgian	D	139	151	179	194	247	263	151	161	193	203	226	228	158	158	250	250	169	169	247	261	243	253	241	253
Codelunghe	D	133	151	179	181	249	263	165	165	193	199	226	228	158	178	250	258	169	177	251	261	245	253	241	251
Corbina	A	135	155	185	189	249	257	151	165	191	195	232	232	162	178	242	250	169	169	239	267	259	261	263	273
Cordenossa	A	135	155	181	189	239	263	141	161	187	193	226	228	158	158	250	258	169	187	251	267	259	259	241	265
Cremin	D	133	143	189	191	239	263	151	151	193	193	226	236	150	168	250	250	169	177	239	247	245	259	273	273
Cuneute	D	143	155	185	185	247	263	141	165	193	203	232	236	162	166	242	250	169	197	231	237	259	261	263	273
Curvin	D	135	151	181	181	247	257	151	161	193	195	232	236	158	178	258	258	169	169	239	261	253	259	253	273
Durriese	D	133	151	185	189	247	257	141	165	193	203	228	228	162	166	248	250	169	177	237	237	245	267	253	253
Forgiarin	A	133	141	181	189	247	257	151	165	193	203	232	240	164	184	250	258	187	187	247	267	243	253	257	273
Fumat	A	133	151	185	191	249	261	161	165	193	199	228	236	168	172	242	260	191	191	231	271	245	257	253	273
Gran Rap Neri	D	155	155	185	189	247	249	141	165	191	193	226	232	176	178	242	250	169	177	247	267	247	261	263	273
Malvasia Istriana	D	143	143	179	179	239	239	161	165	187	187	222	240	138	168	236	254	191	197	257	281	259	259	257	273
Mocula	C	153	155	179	194	239	247	151	165	195	203	226	234	138	150	248	258	177	177	247	251	245	247	251	273
Negrat	D	133	135	185	189	247	257	141	151	195	203	232	232	158	158	250	250	169	169	231	237	253	259	241	253
Nerata	A	135	151	179	181	239	249	169	169	187	199	236	236	166	172	258	258	169	177	237	249	259	267	253	273
Nigrut	D	155	155	185	189	239	247	151	165	193	195	226	228	162	166	242	250	169	169	239	239	245	253	253	273
Palomba Nera	C	135	143	189	194	247	247	151	151	193	195	226	232	150	158	250	256	169	169	239	251	259	259	241	265
Pelena	D	133	145	179	179	239	239	151	165	187	195	240	246	150	166	242	258	177	197	247	257	243	243	253	263
Piccola Nera	C	133	133	181	181	239	249	141	165	195	203	228	234	138	158	242	258	197	197	231	239	243	243	257	273
Piciule	D	133	155	181	189	239	257	141	151	195	195	228	232	166	176	250	258	169	197	251	267	243	259	265	273
Picolit	D	135	139	181	185	247	247	141	161	191	203	232	238	164	168	238	258	169	177	231	237	243	259	241	251
Piculit Neri	A	135	151	179	194	239	263	141	145	193	195	232	246	158	162	248	250	169	187	239	267	259	259	241	265
Pignolo	D	133	155	179	191	247	257	141	167	193	195	226	232	150	158	250	258	177	177	239	247	247	259	241	273
Pinella	D	133	133	179	181	247	249	141	143	203	203	232	234	158	168	242	250	197	197	231	261	243	253	257	273
Polposa	A	133	155	181	185	239	249	165	169	187	199	226	232	162	164	242	250	169	187	237	249	243	261	253	257
Refosco Bianco	D	135	151	185	191	249	257	151	165	195	199	228	232	158	162	250	258	169	169	247	271	259	267	253	263
Refosco dal Peduncolo Rosso	D	133	133	181	189	239	247	141	165	193	193	226	232	158	168	250	258	169	177	237	239	245	259	251	263
Refosco Nostrano	A	151	155	185	185	249	263	165	165	193	199	226	232	162	168	242	250	169	169	237	271	261	267	253	265
Refosco di Rauscedo	D	133	155	179	185	247	263	143	165	193	203	226	226	150	168	250	258	169	169	237	247	245	261	253	263
Ribolla Gialla	A	143	151	179	185	239	249	165	169	195	199	232	234	158	172	236	250	191	197	231	237	259	267	251	253
Ruacit	C	143	151	181	194	247	247	141	159	191	203	226	226	138	176	248	258	191	197	237	249	259	267	263	273
Sagrestana	D	139	151	181	191	239	247	141	169	187	191	226	232	164	164	250	258	177	177	231	249	243	259	241	251
Zimmettraube Blau (Sbulzina)	C	143	143	181	194	239	255	141	169	193	203	226	226	150	158	250	258	177	197	249	263	253	259	253	273
Schioppettino	A	135	139	181	189	239	249	141	169	187	191	228	232	162	176	250	258	187	191	237	239	243	259	257	273
Sciaglin	D	133	135	179	179	247	263	141	161	191	193	228	232	150	162	250	258	169	169	231	237	259	259	257	263
Siora	A	133	139	181	181	239	249	141	165	195	199	228	232	158	178	248	258	169	197	237	239	243	243	265	273
Tazzelenghe	D	151	153	185	185	249	263	151	165	195	199	228	232	162	178	242	250	169	169	237	261	267	271	253	273
Terrano	D	135	155	189	189	247	249	141	143	191	193	226	228	150	158	238	250	169	169	221	237	245	259	251	273
Tintoria Lloyd	A	133	151	189	194	239	247	151	165	193	195	236	238	158	158	244	250	169	191	231	237	253	259	253	273

Tocai Friulano	A	133	151	185	194	239	257	141	151	187	193	228	238	164	166	250	250	169	177	237	251	245	253	241	257
Ucelut	D	133	143	179	189	249	263	151	159	193	199	228	236	158	176	250	254	177	177	237	237	245	259	263	273
Venere	D	135	151	181	185	247	263	141	169	193	203	226	232	164	168	238	258	169	177	231	239	259	259	241	251
Verduzzo Friulano	A	133	151	181	191	239	263	159	169	187	193	226	236	164	178	250	258	177	187	239	249	243	259	241	257
Vinoso Rosso	D	155	155	185	191	247	249	151	165	199	203	226	226	162	162	242	250	169	169	237	267	259	261	253	273
Vubola	A	135	143	185	194	239	257	143	167	187	195	226	228	162	176	242	248	177	187	247	251	259	259	273	273

Additional varieties

Bianchetta Trevigiana	D	135	151	185	189	247	247	151	161	195	203	236	238	150	158	242	244	169	191	247	271	253	259	241	273
Garganega	D	133	143	179	194	249	253	141	165	199	199	226	232	176	178	250	250	177	187	239	251	245	259	251	259
Glera Lunga	D	133	143	179	194	239	247	141	167	187	193	226	246	150	158	248	258	169	197	239	247	243	259	257	273
Goustolidi (Robolla)	C	143	145	179	181	239	249	161	165	195	201	226	234	158	172	236	258	177	197	231	261	245	259	251	273
Heunisch Weiss	C	133	143	179	181	239	249	143	165	195	203	234	240	138	158	236	242	197	197	231	249	243	259	251	273
Honigler	A	143	145	181	194	243	247	151	165	187	203	226	232	138	178	250	258	169	177	251	263	245	259	249	253
Klarnica	A	139	143	189	194	239	249	141	169	187	199	226	228	150	162	250	250	169	191	237	247	243	259	257	273
Marzemino	D	133	133	185	189	239	263	151	165	193	193	226	232	158	168	242	250	169	177	237	239	245	259	241	263
Sauvignon	D	133	151	175	189	239	257	141	151	187	193	228	232	168	172	244	246	169	177	237	239	245	253	241	257
Teroldego	D	137	155	179	185	239	247	161	165	193	193	226	228	158	168	242	254	177	177	231	239	243	245	241	263
Traminer	D	151	151	189	189	243	257	151	161	187	193	232	238	158	168	244	250	169	169	237	239	253	253	241	273
Vela pergola (Volovnik)	A	133	151	179	181	239	247	165	169	187	203	226	240	164	172	250	258	177	187	247	249	243	267	253	257
Vulpea	D	133	135	181	194	239	239	141	141	187	195	228	246	158	176	248	258	187	197	239	251	243	259	257	265

Supporting trios

Raboso Piave	D	135	143	185	189	247	257	151	151	195	195	228	232	158	158	242	250	169	191	237	237	259	259	273	273
Raboso Veronese	D	133	135	185	185	239	257	151	165	193	195	232	238	158	166	242	250	169	169	237	239	245	259	251	273
Marzemina Bianca	D	133	133	185	194	239	253	141	165	193	199	226	238	166	176	248	250	169	177	239	239	245	245	251	253

Glera	D	133	143	179	194	239	247	141	151	187	203	226	246	166	176	248	258	169	197	239	247	243	247	263	265
Vitouska	D	133	145	179	194	239	247	151	165	195	203	226	240	166	176	242	248	177	197	239	257	243	245	253	263
Malvasia Bianca Lunga	D	145	145	179	179	239	253	143	165	195	199	226	240	150	176	242	250	177	177	251	257	243	245	253	257

Pinot	A	137	151	185	189	239	243	151	165	187	193	228	238	158	162	238	244	169	177	221	239	243	253	241	273
Manzoni Bianco	A	151	151	181	185	243	249	143	151	187	203	226	228	138	158	238	242	169	169	231	239	253	253	273	273
Riesling Weiss	A	143	151	181	189	249	257	143	151	193	203	226	234	138	168	242	244	169	197	231	237	253	259	253	273

Table 3S - 14 SNP profiles of the analyzed varieties. In brackets the original names reported in Table 1, if a different prime name is available.

**SNP name by Illumina**	**chr1_12168156_C_T**	**chr11_5406647_A_C**	**chr13_15640723_C_T**	**chr14_4353470_A_G**	**chr14_4947068_G_T**	**chr15_18127737_G_T**	**chr15_18567587_G_T**	**chr16_16198599_A_G**	**chr16_17950801_A_G**	**chr17_126505_A_G**	**chr18_11544918_C_T**	**chr3_5724485_C_T**	**chr8_12906774_C_T**	**chr9_5755442_C_T**
**Chromosome**	**1**	**11**	**13**	**14**	**14**	**15**	**15**	**16**	**16**	**17**	**18**	**3**	**8**	**9**
**Friuli Venezia Giulia varieties**														
Aghedene	TC	TG	TC	GG	TG	TG	AC	AG	AG	AG	CC	TT	TC	AG
Berzamino	TC	TG	TC	AA	TT	TG	AA	AG	AA	AG	TC	TC	CC	AA
Blanchias	TC	TT	TC	GG	GG	GG	CC	AG	AG	GG	CC	TT	CC	AG
Brambana	TC	TT	TC	GG	GG	TG	AC	AG	AA	AA	TC	TC	CC	GG
Cividin	TT	TG	TT	AA	TT	TT	AA	AG	GG	AA	TC	TC	TT	AG
Cjanorie	CC	TT	TT	AG	TG	GG	AA	GG	GG	AG	CC	TC	TC	AG
Cjavalgian	TC	TG	TC	AG	TG	TG	AC	AG	AG	AA	TT	CC	CC	AG
Codelunghe	CC	TG	TT	AG	TT	TG	AA	AA	AA	GG	TT	CC	TC	GG
Corbina	TC	TG	TT	AG	GG	TG	AA	AG	AG	AA	TT	TC	CC	GG
Cordenossa	CC	TG	TC	AG	TG	TG	CC	AG	AG	GG	TC	TC	TC	GG
Cremin	TC	TT	TC	AA	TG	GG	AC	AG	AG	GG	TT	TT	TT	AG
Cuneute	TC	TG	TT	AA	TT	TG	AA	AG	AA	AA	TT	TT	TC	AG
Curvin	TC	TG	TC	AG	TG	TG	AC	GG	AG	AG	TC	TC	TC	GG
Durriese	CC	TG	TC	AG	TG	GG	AA	AG	GG	AG	TC	TT	TC	AG
Forgiarin	TC	TT	TC	AA	TT	TG	AC	AG	AA	GG	TC	TC	TC	AG
Fumat	TC	TT	TC	AA	TT	TG	AA	AG	AA	AG	TC	TC	TC	AG
Gran Rap Neri	TT	TT	TT	AG	GG	TG	AA	GG	AG	AA	TC	TT	TC	GG
Malvasia Istriana	TC	GG	CC	AG	TG	TT	CC	AA	AG	GG	CC	CC	TC	AG
Mocula	TT	TT	TC	AG	TG	GG	AC	AG	AG	AG	CC	TT	TC	AG
Negrat	TC	TT	TT	AA	TG	TT	AC	AA	AG	AA	TC	TC	CC	AG
Nerata	TT	GG	TT	AA	TT	TG	AC	AG	GG	GG	TT	TT	CC	AG
Nigrut	TT	TT	TC	AG	TG	TG	AC	AG	AG	AG	TC	TC	TC	GG
Palomba Nera	TT	TG	TC	GG	TG	TT	AC	AG	AG	AA	TT	TC	TC	AG
Pelena	TT	TG	TT	GG	TG	TG	AA	AG	AG	AG	TC	TT	CC	GG
Piccola Nera	CC	TG	TT	AG	TG	GG	AC	AG	AG	GG	CC	CC	CC	GG
Piciule	CC	TT	TC	AA	TG	TG	AC	AG	AG	AG	TC	TC	CC	GG
Picolit	TT	GG	TT	AG	GG	GG	AA	AA	AA	AG	TC	CC	TC	AG
Piculit Neri	CC	TG	TT	AG	GG	TG	AA	AG	AA	AA	TC	TC	TC	GG
Pignolo	TT	TG	TT	AA	TT	GG	AA	AG	AG	AA	TT	TT	TC	GG
Pinella	TC	TG	TC	GG	GG	TG	CC	AA	AA	AG	CC	TC	CC	GG
Polposa	TC	TT	TT	AA	TT	TG	AA	AA	AA	AA	TT	TC	CC	AA
Refosco Bianco	TC	GG	TT	AA	TT	GG	AA	AG	AG	AA	TC	TC	TC	AA
Refosco dal Peduncolo Rosso	TC	TT	CC	AG	TG	TG	AC	AG	AG	AG	TC	TT	CC	GG
Refosco Nostrano	CC	TG	TT	AA	TT	GG	AA	AG	AG	AA	TC	TC	TC	AA
Refosco di Rauscedo	TC	TG	TT	AG	TT	TG	AA	AA	AA	AA	TT	TC	TC	AA
Ribolla Gialla	TC	GG	TC	AG	TG	GG	CC	AA	AA	AG	CC	CC	CC	AG
Ruacit	TC	GG	CC	GG	TG	GG	CC	AG	GG	GG	TC	CC	TC	GG
Sagrestana	TT	TG	TT	AA	TG	TG	AA	AG	AG	AG	TC	CC	TT	AA
Zimmettraube Blau (Sbulzina)	TT	TG	TC	GG	GG	TG	AA	AA	AG	GG	CC	TT	TT	AG
Schioppettino	TC	GG	TC	AG	GG	GG	AA	AG	AG	AA	CC	CC	TC	AG
Sciaglin	TT	TG	TT	AG	TG	GG	AA	AG	AG	AG	TC	TC	CC	AG
Siora	TC	TG	TC	GG	TG	TT	AC	AG	AG	AG	TC	CC	TC	GG
Tazzelenghe	CC	TG	TT	AA	TG	TG	AC	AG	AG	AA	TC	TC	CC	AG
Terrano	TT	TG	CC	GG	TG	TG	AC	AG	AG	GG	TC	TT	TC	GG
Tintoria Lloyd	TT	TT	CC	AA	TT	GG	CC	AG	AG	AA	CC	TT	TC	GG
Tocai Friulano	TC	TT	TC	AG	GG	GG	AC	GG	GG	AA	TC	CC	TC	AA
Ucelut	TC	TG	TC	GG	TG	TG	AC	AG	AG	GG	TC	TT	TC	GG
Venere	TT	TG	TC	AA	TG	GG	AC	AG	AG	AA	TT	TC	TT	AG
Verduzzo Friulano	TC	TT	TC	AA	TT	TG	AC	AG	AG	AG	TC	TC	TC	AA
Vinoso Rosso	TC	TG	TT	AA	TT	GG	AA	AG	AG	AA	TC	CC	TC	AA
Vubola	TC	TG	TT	AA	TG	GG	AC	AG	GG	AA	TC	CC	TC	AG
**Additional varieties**														
Bianchetta Trevigiana	TC	TT	CC	AG	TG	GG	AC	AG	AA	AA	CC	CC	CC	GG
Garganega	TC	GG	TC	AG	TT	TT	AA	AG	AG	GG	TT	TC	TT	AG
Glera Lunga	TC	TT	TC	AG	TT	TG	AC	AG	AG	AG	TC	TC	TC	GG
Goustolidi (Robolla)	CC	GG	TC	AG	TG	TT	AA	AG	AG	GG	CC	TT	TC	AG
Heunisch Weiss	TC	GG	TC	AG	TG	TG	AC	AA	AG	GG	CC	TC	TC	AG
Honigler	CC	GG	TC	AA	TT	GG	AC	AG	AG	AG	TT	TC	TT	AG
Klarnica	TC	TG	TT	AA	TG	TG	AC	AG	AG	AA	CC	TC	CC	AG
Marzemino	TT	TT	TC	AG	TG	TG	AA	AG	AG	GG	TC	TT	TC	GG
Sauvignon	CC	TT	CC	GG	TG	GG	AC	AG	AG	AA	TC	TC	TC	AG
Teroldego	TT	TG	CC	AA	TT	TG	AA	AG	GG	AG	TT	TC	TT	AG
Traminer	TC	TT	TC	AG	TG	TG	AC	AG	AA	AA	CC	TC	TC	AG
Vela pergola (Volovnik)	TT	TG	TC	AG	TG	TG	CC	GG	AG	GG	TT	TC	TC	AA
Vulpea	CC	TG	TC	AG	TG	TG	AC	GG	AG	AG	TC	TC	CC	GG
**Supporting trios**														
Raboso Piave	TT	TG	TT	AA	TT	GG	AC	AG	GG	AG	CC	TC	TT	GG
Raboso Veronese	TC	TG	TT	AA	TT	TG	AA	AA	AG	AG	TC	TT	TT	GG
Marzemina Bianca	TC	TG	TC	AA	TT	TT	AA	AA	AA	GG	TT	TC	TC	AG
Glera	TC	TT	TT	GG	TG	GG	AA	GG	AG	AG	TT	TC	TC	GG
Vitouska	CC	TG	TT	GG	GG	TG	AA	AG	GG	AG	TC	TC	CC	GG
Malvasia Bianca Lunga	TC	GG	TT	GG	GG	TT	AA	AA	GG	GG	CC	TC	CC	AG
Pinot	TT	TG	CC	GG	GG	GG	AC	AG	AG	AA	TC	TC	TC	AG
Manzoni Bianco	TC	TT	CC	AG	TG	GG	AC	AA	AG	AG	CC	CC	CC	AG
Riesling Weiss	TC	TG	CC	AG	TG	TG	AA	AA	AA	AG	CC	TC	CC	AG

Table 5S - Presumed nSSR genotype of candidate parent 'P2' inferred by Colony. Given the very low number of putative full-sibs compared, hypothetical homozygous loci were integrated with a question mark.

Presumed parent	VVS2		VVMD5		VVMD7		VVMD25		VVMD27		VVMD28		VVMD32		VrZAG62		VrZAG79		ISV2		ISV4		VCMNG4b9	
P2	143	155	226	226?	247	247?	247	259	179	185	247	247?	263	273	193	203	242	258	151	167	169	177	150	166
